# Incidence of cleft Lip and palate in the state of Andhra Pradesh, South India

**DOI:** 10.4103/0970-0358.73443

**Published:** 2010

**Authors:** Srinivas Gosla Reddy, Rajgopal R. Reddy, Ewald M. Bronkhorst, Rajendra Prasad, Anke M. Ettema, Hermann F. Sailer, Stefaan J. Bergé

**Affiliations:** GSR Institute of Craniofacial Surgery, Hyderabad, Andhra Pradesh, India; 1 Radboud University Nijmegen Medical Center, Nijmegen, The Netherlands; 2 Department of Cariology and Preventive Dentistry, Nijmegen, The Netherlands; 3 A. B. Shetty Memorial Dental College and Hospital, Mangalore, Karnataka, India; 4 Department of Oral and Maxillofacial Surgery, Radboud University Nijmegen Medical Center, Nijmegen, The Netherlands; 5 Cleft Children International, Zurich, Switzerland

**Keywords:** Cleft lip & paplate incidence, cleft lip, cleft palate

## Abstract

**Objective:**

To assess the incidence of cleft lip and palate defects in the state of Andhra Pradesh, India.

**Design Setting:**

The study was conducted in 2001 in the state of Andhra Pradesh, India. The state has a population of 76 million. Three districts, Cuddapah, Medak and Krishna, were identified for this study owing to their diversity. They were urban, semi-urban and rural, respectively. Literacy rates and consanguinity of the parents was elicited and was compared to national averages to find correlations to cleft births. Type and side of cleft were recorded to compare with other studies around the world and other parts of India.

**Results:**

The birth rate of clefts was found to be 1.09 for every 1000 live births. This study found that 65% of the children born with clefts were males. The distribution of the type of cleft showed 33% had CL, 64% had CLP, 2% had CP and 1% had rare craniofacial clefts. Unilateral cleft lips were found in 79% of the patients. Of the unilateral cleft lips 64% were left sided. There was a significant correlation of children with clefts being born to parents who shared a consanguineous relationship and those who were illiterate with the odds ratio between 5.25 and 7.21 for consanguinity and between 1.55 and 5.85 for illiteracy, respectively.

**Conclusion:**

The birth rate of clefts was found to be comparable with other Asian studies, but lower than found in other studies in Caucasian populations and higher than in African populations. The incidence was found to be similar to other studies done in other parts of India. The distribution over the various types of cleft was comparable to that found in other studies.

## INTRODUCTION

Oro-facial clefts, particularly cleft lip with (CLP) or without (CL) cleft palate and cleft palate alone (CP) are a major public health problem affecting 1 in every 500 to 1000 births worldwide.[1,2] A child is born with a cleft somewhere in the world every 2 minutesaccording to a WHO study published in 2001.[3] In India alone the number of infants born every year with CLP is 28,600, which means 78 affected infants are born every day, or 3 infants with clefts born every hour.[4]

India is the second most populated country in the world with a population of 1.02 billion.[5] For its administration it is divided into 28 states and 7 union territories. Each state is governed by an elected local government, while the union territories are governed by the Government of India directly through its representatives. Andhra Pradesh state is in the south east of India.

The purpose of this study was to find out the incidence of cleft lip and palate in Andhra Pradesh. This study was carried out by a high volume center in the state of Andhra Pradesh in association with the Government of Andhra Pradesh. The reason for the government’s involvement with this project was to find out its impact on the health delivery system so that effective treatment could be given.

## STUDY DESIGN

Andhra Pradesh has a population of 76 million and is divided into 23 districts. Hyderabad is its capital city.

Three districts were chosen for this study Cuddapah, Medak and Krishna. They were chosen for this study for their socio-economical diversity.

Cuddapah district is a predominantly rural district that is drought prone and under developed with the lowest per capita income in the state. The population has very little access to health care.

Medak is a semi-urban district. It is also an arid district but the population here has access to good health care because of its proximity to Hyderabad.

Krishna district has the highest per capita income after the capital Hyderabad. It is an urban district where the population can afford health care.

This study was conducted in 2001. The survey was conducted in collaboration with the administrative head of each district, known as the District Collector or Magistrate, through a unique program called the ‘*Janmabhoomi Programme*’.

During this programme the government did a detailed health survey of each of the three districts Cuddapah, Medak and Krishna, where a medical questionnaire is filled in by each resident of the village/town. Cleft defects were included in the questionnaire in the form of one question which was, do you know anyone in your village/neighborhood that has a facial defect? If the answer was yes, then the local Primary Health Worker would identify the person with the cleft defect and investigate whether the cleft was a CL, CLP or CP. If the cleft was a CL or CLP then the side of the cleft would be noted. The parents’ literacy and consanguinity would be noted.

Simultaneously, data was also collected through the District Medical and Health Officer (DMHO) and the District Education Officer (DEO). The DMHO obtained information of all clefts born or reported through primary health workers who are there at primary health centers, which are established in every three villages and in every town. The DEO obtained the information through schools, which are established in every village and town. All the data recorded was entered into a database for further correlation.

The literacy rates of the parents were used as a marker because it is very difficult to obtain the exact per capita income of parents of children born with cleft defects living in rural areas, who depend on agriculture for their earnings. Agriculture in India is seasonal and yearly income fluctuates from year to year and therefore an accurate reading for income is not readily available. The literacy of a person was thought to correspond better to the socio-economic status of that person. Income from agriculture in India is exempt from income tax, and people completely dependent on agriculture need not file the details of their income. This means that all government data on income in the rural areas of India are hypothetical and not exact.

Consanguinity is a widely practiced ritual in Andhra Pradesh. Marriages up to one degree of separation were taken to be a consanguineous relationship i.e. between a girl and her maternal uncle or between the girl and her maternal uncle’s son or her fraternal aunt’s son. Therefore consanguinity was noted of the parents to establish a correlation between the two if any.

The age at the time of the survey and sex of the affected child were also noted. The age at the time of the survey was noted so that all children below 1 year of age could be counted towards the live births in the district for that year.

To estimate the incidence of clefts based on gender, parents’ literacy and consanguinity, background variables with a known distribution in the general population was needed. National census information for gender and literacy was available. To obtain the background variables for consanguinity a small cross-section study was undertaken (*N*=100) in each of the three districts.

The population, distance from Hyderabad and literacy rates of Andhra Pradesh and the three districts are given in Table 1.

**Table 1:** Demographic information on state and district level

	*Andhra Pradesh*	*Cuddapah district*	*Medak district*	*Krishna district*
Population	76,210,007	2,573,480	2,662,290	4,218,410
Distance from Hyderabad (km)		300	50	250
Literacy rate (%)	61.11	64.02	53.24	69.91

The number of patients with cleft defects in the three districts were Cuddapah 556, Medak 490 and Krishna 376. The age at the time of the survey and sex of the patient are given in Table 2.

**Table 2:** Age and sex of patients

	*Age at the time of survey*			*Sex*	
	*<1 year*	*1–16 years*	*>16 years*	*Male (%)*	*Female (%)*
Cuddapah district	75	390	91	405 (73.1)	405 (73.1)
Medak district	57	345	88	313 (63.8)	177 (36.2)
Krishna district	71	241	64	218 (58.1)	158 (42.5)

There was a male predominance for the cleft defects in all the districts under study [Table 2]. Literacy rates of the parents of affected patients showed that Krishna district statistically had the highest literacy rates as compared to other districts [Table 3].

**Table 3:** Consanguinity and education levels of the parents

	*Consanguinity of parents*		*Education levels of the parents*				
	*Consanguineous*	*Nonconsanguineous*	*No education (%)*	*Primary school (%)*	*High school (%)*	*Graduate (%)*	*Post graduate (%)*
Cuddapah district	324	232	698 (76.7)	171 (18.8)	25 (2.8)	14 (1.5)	2 (0.2)
Medak district	243	247	573 (63.5)	262 (29.1)	46 (5.1)	18 (2.0)	3 (0.3)
Krishna district	155	221	292 (40.1)	251 (34.5)	151 (20.7)	24 (3.3)	10 (1.4)

With regard to consanguineous marriages Cuddapah had the highest percentage within the patient group compared to the other two districts [Table 3].

There was a high incidence of CLP in all three districts followed by CL. The incidence of isolated CL was very low [Table 4].

**Table 4:** Type and side of cleft per district

	*Cuddapah district (%)*	*Medak district (%)*	*Krishna district (%)*
Isolated cleft lip	167 (30)	156 (32)	139 (37)
Cleft lip and palate	368 (66)	319 (65)	226(60)
Unilateral	422 (79)	370 (78)	292 (80)
Left	262 (62)	244 (66)	184 (63)
Right	160 (38)	126 (33)	108 (37)
Bilateral	113 (21)	105 (22)	73 (20)
Isolated cleft palate	16 (3)	12 (2)	9 (2)
Craniofacial cleft	5 (1)	3 (1)	2 (1)

There was a higher prevalence of unilateral clefts to bilateral clefts. In unilateral clefts there was a higher prevalence of left-sided clefts as compared to right sided ones [Table 4].

We calculated the incidence of clefts in each district by registering the total number of live births in each district in 2001 and dividing it by the number of children who had clefts and were under 1 year in the same district.

The incidence of clefts in Andhra Pradesh was calculated by defining the average of the incidence of clefts in the three districts. The number of live births in Andhra Pradesh during the same period was 1,666,000 (Census of India 2001). Assuming the incidence in the state as 1.09 out of 1000 live births, 1830 children were born with cleft defects in the state of Andhra Pradesh in 2001 [Table 5].

**Table 5:** Incidence of clefts per district

	*Cuddapah District*	*Medak District*	*Krishna District*	*Andhra Pradesh State*
Live births	65,562	49,504	69,741	184,807
Cleft defects	75	57	71	203
Incidence	1.14 in 1000 live births	1.15 in 1000 live births	1.01 in 1000 live births	1.09 in 1000 live births

The relation between incidence of clefts and background variables was also calculated. For gender, the national census information shows that the ratio of males to females is 1000 : 993 (Census of India 2001). This information is with regard to the national ratio as information on a district level is not available. For literacy rates, using an education level of primary school or higher as a definition of “literate”, the percentages for the districts were Cuddapah 64.5%, Medak 53.2% and Krishna 69.9%(Census of India 2001). Based on consanguinity, the prevalence in a small cross-sectional study of 100 couples in each of the three districts was Cuddapah 21%, Medak 12% and Krishna 9%. To convert the percentages into absolute numbers for the background variables for gender and literacy it was arbitrarily chosen to be 1000.

The odds ratio for a child being born with a cleft in relation with gender is 2.50, 1.65 and 1.29 for the districts of Cuddapah, Medak and Krishna, respectively. With regard to parents’ illiteracy the odds ratios are 5.85 for Cuddapah, 1.98 for Medak and 1.55 for Krishna. Consanguinity is 5.25, 7.21 and 7.09 for the three districts, respectively. All 9 odds ratios are statistically significantly above 1, as can be seen from the 95% confidence intervals [Table 6].

**Table 6:** Relation between clefts and gender, literacy and consanguinity per district

	*Cuddapah district*			*Medak district*			*Krishna district*		
	*Case*		*Ctrl*	*Case*		*Ctrl*	*Case*		*Ctrl*
Gender									
Male	405		517	313		517	218		517
Female	151		483	177		483	158		483
Odds ratio		2.50			1.65			1.29	
95% CI for OR		[1.99 …3.15]			[1.32…2.07]			[1.01…1.64]	
Literacy									
No	430		360	305		468	150		301
Yes	131		640	175		532	226		699
Odds ratio		5.85			1.98			1.55	
95% CI for OR		[4.61…7.44]			[1.58…2.49]			[1.26…1.90]	
Consanguinity									
Yes	232		79	247		88	221		91
No	324		21	243		12	155		9
Odds ratio		5.25			7.21			7.09	
95% CI for OR		[3.12…8.84]			[3.80…13.70]			[3.42…14.71]	

## DISCUSSION

This study was conducted in 2001. The reason for a delay in publishing these findings is that the study of incidence of clefts was part of a much larger programme. This programme was a statewide developmental programme where the Government of Andhra Pradesh was reaching out to 80 million people of the state. The districts involved with this study have a population in excess of 9.45 million. Since most of the data, including the data the Government of Andhra Pradesh was collecting, was being collected for the first time, sorting out and collating the data took time.

There are also some drawbacks to a study of this size. Firstly, the study had to be simplified such that very few questions had to be asked by the interviewers as other programmes were being jointly run by the government. Secondly, the staff interviewing the subjects were not trained doctors and therefore could only be trained to identify a cleft, and therefore a detailed report on conditions that might have contributed to the cleft could not be included in the study.

Although three districts were selected to reflect the socio-economical diversity of the Andhra Pradesh, the generalization from the district to the state level is hypothetical. However, given the size of the state, its population and resources available, it the best way one can shed some light on a topic on which, so far, very little information is available.

While calculating the incidence of cleft based on the data collected, it was agreed that those children who were less than one year of age would be included in the census as a simultaneous census to see that the total number of live births in that region was concurrently going on. We do accept that there might be misrepresentation of children that might have died during the year. However, we accepted this method because registry for births and deaths is not very accurately maintained in large parts of rural areas of the state.

A review of studies for incidence of cleft lip and palate shows that there is no particular trend in different parts of the world. A WHO study published as Global Strategies to Reduce the Health Care Burden of Craniofacial Anomalies in 2000 details the incidence in 13 countries and the incidence varies from 0.22 to 1.67 per 1000 live births.[3]

Incidence of oral-facial clefting show ethnic variation. It is generally thought that populations of Asian or Native North American descent have the highest incidence, with Caucasian populations having intermediate incidence and African populations having the lowest incidence.[6]

In studies conducted on Caucasian population, the incidence of clefts in Northern Ireland was found to be 1.28 for every 1000 live birth.[7] The incidence of cleft defects in Stockholm County in Sweden was found to be 2.0:1000 live birth.[8]

In studies conducted in Latin American population, a study in Northeast Mexico showed an incidence of clefts to be 1.1:1000 live birth,[9] while a study carried out in an African population in Nigeria showed a birth rate of cleft anomalies at 0.4:1000 live birth.[10]

In Asia, a study of a Han Chinese population in Shanghai, China[11] showed an incidence of 1.12 per 1000 live births. A study of a native Filipino population reported that the incidence was higher at 1.54 in 1000 live births.[12] A study in Iran showed an incidence of 1.03:1000 live birth.[13] Our study showed an incidence of 1.09 in 1000 live births. In India meta-analysis of 25 early studies from 1960 to 1979 involving 407,025 births showed 440 births with CLP and 25 births with CP with an incidence of 1.08 and 0.23 in 100 live births, respectively.[4]

Most studies, including ours, report a male predominance in the sex ratio in cleft lip and palate patients and a female predominance in patients with cleft palate defects also.[14–16] However one district in our study showed an unusually high male predominance for clefts. This discrepancy needs to be further investigated for any extraneous circumstances. Male predominance for cleft lip and palate was also confirmed by the odds ratio which determined that there was a greater possibility of a male child being born with a cleft lip and palate.

Most studies give a ratio between unilateral and bilateral cleft lips to be predominantly favoring unilateral cleft lips.[15,17,18] We found 79% of the cleft lip defects were unilateral in nature. It is also widely accepted that left-sided unilateral clefts are more common than right-sided unilateral cleft lips,[10,14] which is supported by this study. Of the unilateral cleft lips in our study 64% of were left sided.

The type and extent of cleft defects vary according to race. In study published on a Caucasian population, the prevalence of CL was 25%, CLP 50%, and CP 25%[19] (Croen LA 1998). A study on an African population, done in Nigeria showed prevalence to be CL 49%, CLP 32% and CP 19%.[10] Our study showed a prevalence of CL to be 33%, CLP 64%, and CP 3%. The reason for the low percentage of CP could be due to under reporting of the problem. Cleft palate may be undiagnosed at birth and could have been missed in the evaluation of patients.

As stated earlier consanguinity is a widely practiced ritual in Andhra Pradesh. Our study shows that consanguinity of parents is a major risk factor for cleft formation. This study highlights the regressive nature of this practice.

We found a significant differentiation in cleft birth rates between urban and rural areas which are in contrast with the Chinese study.[16] We found a strong correlation between illiteracy and clefts in our study. We also found that illiteracy rates were higher in rural areas in the state. We feel, and this was confirmed by the study, that poorer sections of society are more likely to be illiterate and the odds ratio of a cleft being born to illiterate parents is considerably higher. Malnutrition could also be a possible cause of clefts in undernourished parents of a cleft child. Additionally it should be noted that a relation between illiteracy and consanguinity is likely, with a higher percentages of consanguinity in illiterate populations. Therefore, a part of the relation between illiteracy and cleft birth rates might be due to confounding between illiteracy and consanguinity. Data to check this is currently not available. This implies that the relation between illiteracy and cleft birth rate needs more study to assess its true size and understand the biological mechanism.

## CONCLUSION

Our study showed an incidence of 1.09 in 1000 live births, which was significantly less than the Caucasian and Filipino population studies and significantly higher than the African population study. It was however comparable to other Asian studies like those done in China, Iran and particularly India. It was also comparable to study done in Mexico.

Three diverse districts of Andhra Pradesh were chosen to represent the state. This exercise could be used in other districts to accurately find the incidence of clefts.

Data sources may influence or bias the results. Thus precise documentations of birth and death registry will help evaluate the true values of incidence.
